# Papillomacular bundle defect (PMBD) in glaucoma patients with high myopia: frequency and risk factors

**DOI:** 10.1038/s41598-023-48687-0

**Published:** 2023-12-11

**Authors:** Min Gu Huh, Young In Shin, Yoon Jeong, Young Kook Kim, Jin Wook Jeoung, Ki Ho Park

**Affiliations:** 1 Department of Ophthalmology, Seoul National University Hospital, Seoul, Republic of Korea; 2 Department of Ophthalmology, Seoul National University College of Medicine, Seoul, Republic of Korea

**Keywords:** Diseases, Risk factors

## Abstract

Little is known about the papillomacular bundle defect (PMBD) in glaucoma. As such, we investigated the frequency of PMBD in glaucoma patients with high myopia, and its risk factors. In this retrospective, cross-sectional study, retinal nerve fiber layer (RNFL) defect was analyzed in 92 glaucomatous eyes with high myopia (axial length of 26.0 mm or more or an average spherical value of − 6.0 diopters or less). After dividing them into two groups with and without PMBD, the clinical characteristics of the groups were compared and analyzed. The mean age of the patients was 52.1 ± 10.5 years, and there were 53 males and 39 females. PMBD were observed in 55 eyes (59.8%). There was no significant intergroup difference in baseline or follow-up intraocular pressure (IOP). Parapapillary atrophy (PPA)-to-disc-area ratio (OR 3.83, CI: 1.58–10.27, p = 0.010), lamina cribrosa defect (LCD; OR 2.92, CI: 1.14–8.13, p = 0.031) and central visual field defect (CVFD; OR 3.56, CI: 1.38–9.58, p = 0.010) were significantly associated with the PMBD.

.

## Introduction

Glaucoma is the second most frequent cause of blindness in the world. It proceeds by disturbance of axoplasmic flow in the retinal ganglion cells (RGCs) leading to retinal nerve fiber layer defect (RNFLD) and corresponding visual field (VF) loss^1, 2^.

Myopia prevalence is increasing worldwide, especially in East Asian countries, where the rate of new cases is exceedingly high^3–7^. Myopia is a significant glaucoma risk factor. Myopia-related structural change is correlated with mechanical stress exerted on the optic nerve head (ONH), which in turn has been proposed as the cause of glaucomatous RGC damage. Additionally, myopic refraction is now known to have a significant relationship with depression of the cecocentral VF in glaucoma^8, 9^.

By the end stage of glaucoma, normally the papillomacular bundle (PMB) area is defective. Papillomacular bundle defect (PMBD) tends to be correlated with long axial length, large optic disc and normal-tension glaucoma diagnosis^10^. However, not much more is known about PMBD in cases of highly myopic glaucoma^11–13^. Therefore, in the present study, we investigated PMBD frequency in highly myopic patients with glaucoma and analyzed PMBD risk factors and clinical characteristics.

## Results

### Subject demographics

Ninety-two (92) patients were included in the final analysis. Their average age was 52.1 ± 10.5 years, the mean spherical equivalent was − 8.1 ± 1.7 diopters, and the mean axial length was 26.6 ± 1.0 mm. The mean value of average GCIPL thickness was 62.0 ± 8.2 μm, and the mean value of average RNFL thickness was 65.6 ± 9.9 μm. The mean deviation (MD) was − 8.6 ± 6.0 dB, and the mean visual field index (VFI) was 77.9 ± 17.6%. Among the glaucoma patients with high myopia, PMBD was observed in 55 cases (59.8%). There was a significant difference in average GCIPL thickness and temporal RNFL thickness between the PMBD and non-PMBD groups (Table 1). In the group with PMBD, the mean PPA-to-Disc-area ratio was 0.90 ± 0.65, significantly larger than in the group without PMBD (0.55 ± 0.37). Lamina cribrosa defect (LCD) was observed in 37 eyes (40.2%) of the total patient group, 27 eyes (49.1%) of the group with PMBD and 10 eyes (27.0%) of the group without PMBD (Table 1). Central visual field defect (CVFD) was observed in 56 eyes (60.9%) of the total patient group, 40 eyes (72.7%) of the group with PMBD, and 16 eyes (43.2%) of the group without PMBD (Table 1).

**Table 1:** Descriptive statistics of study population.

	Group with PMBD (N = 55)	Group without PMBD (N = 37)	*p*-value
Age (years)	51.5 ± 9.7	53.1 ± 11.9	0.456
Sex (female, %)	24 (43.6%)	15 (40.5%)	0.936
Hypertension (n, %)	5 (9.1%)	6 (16.2%)	0.480
Diabetes mellitus (n, %)	2 (3.6%)	4 (10.8%)	0.349
Spherical equivalent (D)	− 8.2 ± 1.7	− 8.1 ± 1.8	0.797
Axial length (mm)	26.5 ± 1.0	26.6 ± 1.0	0.794
OCT parameter			
Average GCIPL thickness (µm)	60.4 ± 8.4	64.3 ± 7.5	**0.025**
Average RNFL thickness (µm)	64.3 ± 9.9	67.7 ± 9.6	0.101
Superior (µm)	77.0 ± 17.0	78.5 ± 15.4	0.654
Inferior (µm)	66.6 ± 14.5	68.1 ± 16.2	0.663
Nasal (µm)	58.9 ± 8.8	60.8 ± 9.3	0.316
Temporal (µm)	54.0 ± 15.1	63.7 ± 14.7	**0.002**
Visual field parameter			
Mean deviation (dB)	− 9.0 ± 6.4	− 7.9 ± 5.4	0.394
Pattern standard deviation (dB)	8.9 ± 4.2	8.6 ± 4.2	0.710
Visual field index (%)	76.1 ± 18.7	80.6 ± 15.6	0.222
Lamina cribrosa defect (n, %)	27 (49.1%)	10 (27.0%)	0.057
Disc tilt direction	− 8.8 ± 16.8	− 7.0 ± 12.4	0.553
PPA-to-disc-area ratio	0.90 ± 0.65	0.55 ± 0.37	**0.004**
Disc hemorrhage (n, %)	8 (14.5%)	3 (8.1%)	0.544
Central visual field defect (n, %)	40 (72.7%)	16 (43.2%)	**0.017**

Data are recorded as mean ± standard deviation.

*PMBD* papillomacular bundle defect, *OCT* optical coherence tomography, *GCIPL* ganglion cell-inner plexiform layer, *RNFL* retinal nerve fiber layer, *PPA* parapapillary atrophy.

The P values in the table refers to the comparison between group with PMBD and without PMBD.

### Intraocular pressure (IOP) follow-up in highly myopic glaucoma

The mean baseline IOP for the total patient group was 16.4 ± 3.8 mmHg, and during follow-up, the mean maximum IOP was 17.5 ± 3.0 mmHg, the mean minimum IOP was 10.5 ± 1.6 mmHg, and the mean percentage of IOP reduction from the baseline IOP was 16.8 ± 13.9%. There was no difference in IOP readings, even when the total patient group was divided by the presence or absence of PMBD (Table 2).

**Table 2:** Intraocular pressure (IOP) of groups with papillomacular bundle defect (PMBD) and without PMBD.

	Group with PMBD	Group without PMBD	*p*-value
Baseline IOP (mmHg)	16.1 ± 3.76	16.6 ± 3.79	0.484
Maximum follow-up IOP (mmHg)	17.2 ± 3.00	17.7 ± 2.89	0.493
Minimum follow-up IOP (mmHg)	10.4 ± 1.62	10.6 ± 1.63	0.523
Mean follow-up IOP (mmHg)	13.6 ± 1.69	13.1 ± 1.92	0.237
IOP reduction (%)	17.3 ± 12.3	16.6 ± 14.9	0.809

Data are recorded as mean ± standard deviation.

*PMBD* papillomacular bundle defect, *IOP* intraocular pressure.

The P values in the table refers to the comparison between group with PMBD and without PMBD.

IOP reduction (%) was calculated by the following formula. Baseline IOP (mmHg) − Mean follow-up IOP (mmHg)/Baseline IOP (mmHg) × 100 (%).

### Factors associated with PMBD in highly myopic glaucoma

**Figure 1:**
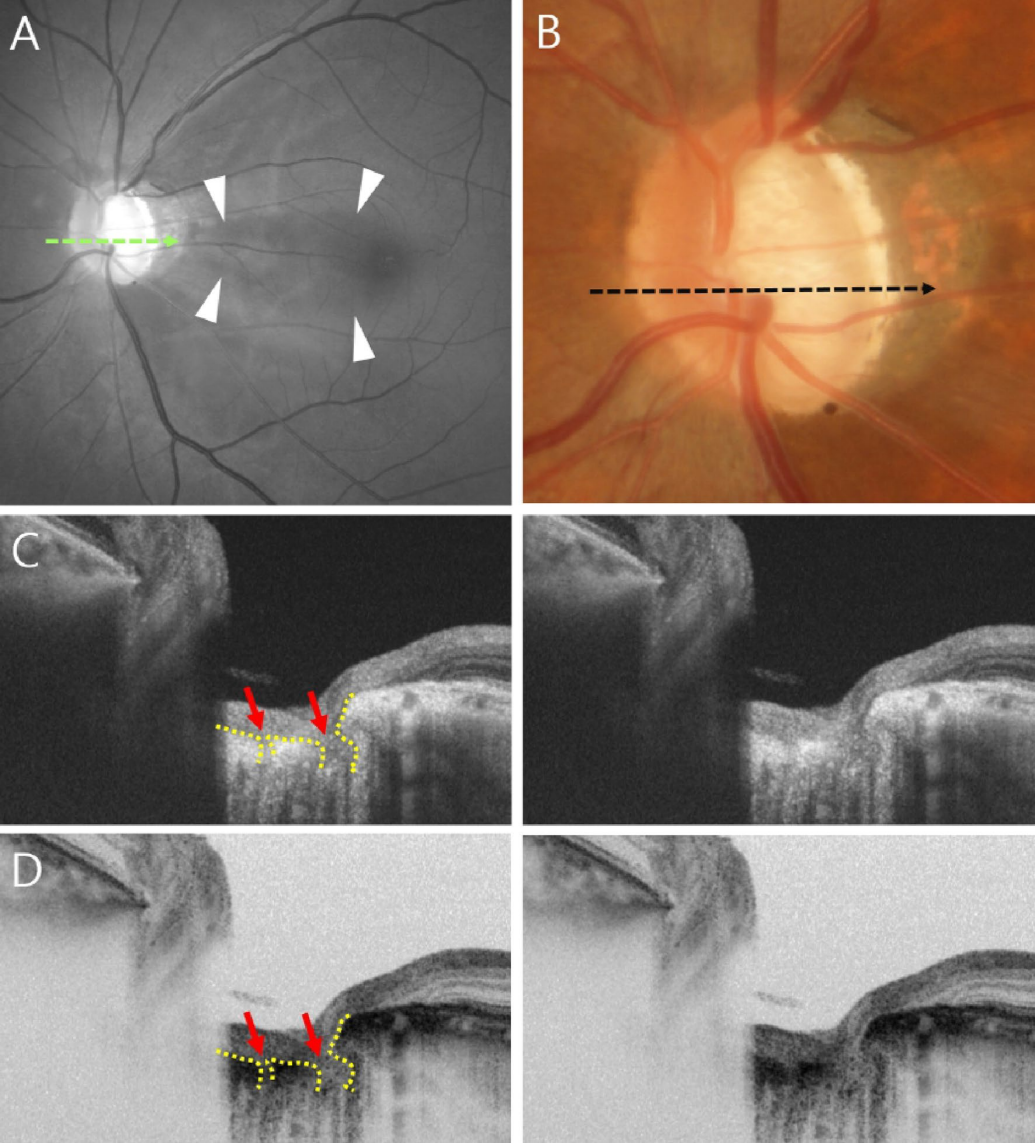
Representative case of lamina cribrosa defect (LCD) associated with papillomacular bundle defect (PMBD). (**A**) A PMBD (within arrowheads) in 55-year-old man with highly myopic glaucoma (spherical equivalent − 6.75 D, axial length 26.52 mm, mean deviation [MD] − 12.66 dB). (**B**) Optic disc photograph. (**C**) LCD (red arrows) in horizontal B-scan image. (**D**) B-scan image that reverses black and white image in (**C**). The B-scan images (**C**,**D**) of the LCD correspond to the green dotted line in (**A**) and the black dotted line in (**B**).

**Figure 2:**
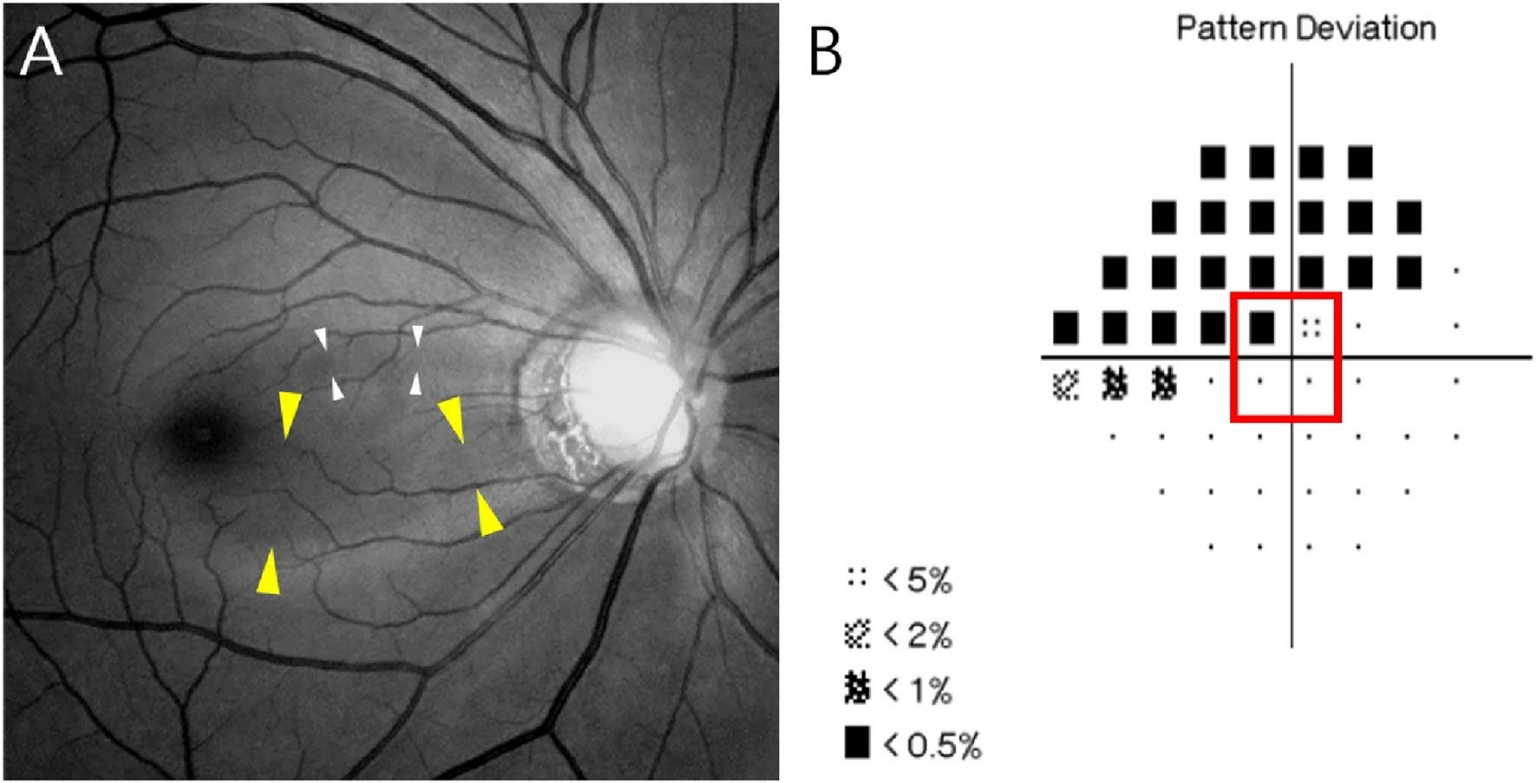
Representative cases of central visual field defect (CVFD) associated with PMBD. (**A**) Retinal nerve fiber layer defects (white and yellow arrowheads) involving PMB area in 45-year-old woman with highly myopic glaucoma (spherical equivalent − 6.125 D, axial length 26.45 mm, MD − 11.48 dB). (**B**) CVFD (red square) at innermost 4 points on pattern deviation of 24–2 visual field (VF) test. This CVFD is thought to correspond to the larger PMBD indicated by the yellow arrowheads in (**A**).

In the univariate logistic regression analyses investigating associated factors, PMBD was significantly associated with the larger PPA-to-Disc-area ratio (OR 3.73, CI 1.94–8.79, p = 0.009), and the presence of LCD (OR 2.60, CI 1.08–6.60, p = 0.036) and CVFD (OR 3.14, CI 1.32–7.69, p = 0.010) (Table 3). Also in the multivariate logistic regression analyses investigating associated factors, PMBD was significantly associated with the larger PPA-to-Disc-area ratio (OR 3.83, CI 1.58–10.27, p = 0.010), and the presence of LCD (OR 2.92, CI 1.14–8.13, p = 0.031) (Fig. 1) and CVFD (OR 3.56, CI 1.38–9.58, p = 0.010) (Fig. 2) (Table 3).

**Table 3:** Univariate and multivariate logistic regression analysis for PMBD.

	Univariate		Multivariate	
	Odds ratio (95% CI)	*p*-value	Odds ratio (95% CI)	*p*-value
Age	0.98 (0.95–1.02)	0.452		
Sex	1.14 (0.49–2.67)	0.768		
Diabetes mellitus	0.31 (0.04–1.69)	0.191	0.25 (0.03–1.98)	0.199
Hypertension	0.52 (0.14–1.85)	0.307		
Baseline IOP	1.04 (0.93–1.17)	0.480		
Mean follow-up IOP	1.16 (0.91–1.49)	0.237	1.07 (0.81–1.43)	0.611
IOP reduction	1.00 (0.97–1.03)	0.807		
Mean deviation	0.97 (0.90–1.04)	0.391		
Spherical equivalent	0.97 (0.75–1.24)	0.819		
Lamina cribrosa defect	2.60 (1.08–6.60)	**0.036**	2.92 (1.14–8.13)	**0.031**
Disc tilt direction	1.01 (0.98–1.04)	0.551		
PPA-to-disc-area ratio	3.73 (1.94–8.79)	**0.009**	3.83 (1.58–10.27)	**0.010**
Disc hemorrhage	1.93 (0.52–9.30)	0.357		
Central visual field defect	3.14 (1.32–7.69)	**0.010**	3.56 (1.38–9.58)	**0.010**

*CI* confidence interval, *IOP* intraocular pressure, *PPA* parapapillary atrophy.

## Discussion

This study showed that PMBD is associated with CVFD; other studies, meanwhile, have shown a strong relationship between PMBD and visual acuity and a causal relationship between PMBD and diminished vision in patients with glaucoma^19, 20^. Certainly, the PMB is a structure that plays an important role in central vision, and as such, it is expected that PMBD can affect the quality of life of patients quite significantly. Thus, the role of PMBD in ocular diseases has been a major concern of researchers. Glaucoma patients with PMBD have been known to suffer central scotoma in the early disease stage^21, 22^, even though the PMB area usually becomes defective at the end stage of glaucoma^23^. Kimura et al.^24^ reported a significant association between high myopia and the nearest RNFL defect involving the PMB in early-glaucomatous eyes. PMBD was found in 55.7% of early-glaucoma patients with high myopia, but only 27.2% of patients with non-highly myopic eyes, a significant difference (p = 0.002). Our results are consistent: PMBD was found in fully 59.8% of highly myopic glaucoma patients.

It is well known that PPA frequency and size are related not only to glaucoma but also to myopia. Miki et al.^25^ reported that the beta zone was positively correlated with both axial length (P = 0.039) and glaucoma (P = 0.011). Jonas et al.^26^ noted that PPA as a whole and both the alpha and beta zones were significantly larger, and that the beta zone was significantly more frequent in their glaucoma group than in normal individuals. The patient group in the present study included a high-myopia group, which would be expected to include patients with an overall large PPA area. However, even in this specific patient group, significant differences in PPA-to-Disc-area ratio were found when dividing the population based on the presence or absence of PMBD. As larger PPA-to-Disc-area ratio signals greater myopic deformation of the optic disc and peripapillary tissue, there must have been greater mechanical stress in the region of deformation. This mechanical stress and tissue susceptibility may be associated with PMBD in the corresponding location. Therefore, the higher the myopia, the larger the PPA area that will be found; and notably too, the results of this study suggest that larger PPA area may be related to risk of RNFL thinning in the PMB area.

In glaucoma pathogenesis, the LC is known to be the primary site of damage to RGC axons^27–30^ and moreover, LC defect is associated with RNFL loss^31^. In the present study, LCD was found in 40.2% of highly myopic glaucoma patients, and 72.9% of glaucoma patients with LCD had PMBD. The comparative analysis of the non-PMBD group showed that the risk factor for development of PMBD was LCD. According to a previous study^32^, LCD (i.e., acquired optic nerve pits) at the temporal edge was confirmed in 31.6% of high-myopia patients. It is believed that this area might be vulnerable to structural damage in cases of high myopia.

Recent evidence seems to indicate that CVFD is not just a characteristic of glaucoma in its late stages, but is actually a feature of early disease^33–36^. Given that this region has the highest RGC density^37^ and is also vital for everyday visual function^38^, a CVFD that occurs close to fixation might impact patients’ essential activities of daily life^39^. Even in patients who have relatively small CVFDs, vision-related quality of life can be significantly affected^40^. Araie et al.^8^ found higher myopia to be significantly associated with VF damage that was just temporal and inferior to the fixation point, in advanced glaucomatous eyes. Mayama et al.^9^ reported that CVFD in a highly myopic primary open-angle glaucoma (POAG) eye showed a gradual increase with glaucoma progression, whereas in an emmetropic normal tension glaucoma (NTG) eye, the central VF was relatively well preserved despite the glaucoma progression. We found CVFD in 62.0% of glaucoma patients with high myopia, and were able to confirm that it was significantly associated with the presence or absence of PMBD.

The present study has several limitations that must be noted. First, the subjects had been recruited from one tertiary referral hospital, and all were Korean. Second, this study was cross-sectional in design, and PMBD could be more prevalent in a longitudinal study. Also, proving a causal relationship between LCD and PMBD was, from the developmental point of view, difficult. Third, RNFL defect detection may have been affected by tessellated fundus. Even in those eyes with tessellated fundus, however, the inter-observer agreement for detection of RNFL defect and its meiasurement was acceptable. Fourth, this study is limited by OCT’s intrinsic properties, especially the fact that its sensitivity and signal strength decrease with depth. Although SS-OCT allows for deeper penetration of light for better delineation of more posterior structures of the ONH and ocular wall, its penetration depth remains, nonetheless, a limitation. Although we identified disc hemorrhage (DH) during follow-up, it is possible that instances that had occurred before follow-up or between follow-up intervals were missed. Therefore, it is possible that the number of DHs in the total patient group was low; thus, additional research based on a longer follow-up and a larger number of disc photo scans is necessary in order to more fully investigate the relationship between PMBD and DH. Fifth and finally, this study has a limitation in that evaluation of the central VF as related to PMBD was performed only with the central 24–2 test. The central 10–2 test, significantly, can be prospectively performed and the relationship between central 10–2 results and PMBD will be an important focus in future studies. Even so, reports have shown that the 24–2 and 10–2 tests’ utilities for detection of VF loss in evaluating central vision are not significantly different^41^, and that the results of the 10–2 test can be predicted via the 24–2 test^42^.

In conclusion, Papillomacular bundle defect (PMBD) was observed in 59.8% of glaucoma patients with high myopia, and was correlated with larger PPA-to-Disc-area ratio and the presence of LCD and CVFD. Therefore, for highly myopic glaucoma patients, the presence of PMBD and/or LCD possibly affecting the central VF should be carefully evaluated.

## Methods

This was a single-center retrospective study performed at Seoul National University Hospital. The study was approved by the Institutional Review Board of Seoul National University Hospital, Seoul, Korea (IRB number: 2209–006-1354), which adhered to the tenets of the Declaration of Helsinki. Due to the retrospective nature of the study, Institutional Review Board of Seoul National University Hospital waived the need of obtaining informed consent.

### Study participants

All of the study participants had visited the Glaucoma Clinic of Seoul National University Hospital, Seoul, Korea, between January 2015 and January 2022. The participants were consecutively enrolled based on a retrospective review of their medical records. On the initial visit to the Clinic, all underwent a full ophthalmic examination entailing a medical history review, best-corrected visual acuity measurement, slit-lamp biomicroscopy, Goldmann applanation tonometry (Haag-Streit, Koniz, Switzerland), gonioscopy, funduscopic examination (90 diopter lens), stereoscopic optic disc photography, red-free retinal nerve fiber layer (RNFL) photography, circumpapillary retinal nerve fiber layer (cpRNFL) thickness measurement, macular ganglion cell-inner plexiform layer (GCIPL) thickness measurement, and optic nerve head (ONH) parameter measurement by Cirrus spectral-domain optical coherence tomography (SD-OCT) (Carl Zeiss Meditec, Dublin, CA, USA), lamina cribrosa (LC) imaging by swept-source optical coherence tomography (SS-OCT) (Topcon, Inc., Tokyo, Japan) and central 24–2 threshold testing of the Humphrey visual field (HVF) (HFA II; Humphrey Instruments Inc., Dublin, CA, USA).

Glaucomatous eyes were defined by their characteristic localized or diffuse neuroretinal rim thinning of the optic disc (on stereo disc photography) or by the presence of RNFL defect (on red-free fundus imaging). Glaucomatous VF defect was defined as follows: (1) a 3-point cluster of lower than 5% probability in a location typical for glaucoma of a pattern deviation map, at least 1-point cluster with a lower than 1% probability; (2) glaucomatous hemifield test results outside the normal limits; or (3) a pattern standard deviation of more than 95% of the normal limits, as confirmed on at least 2 reliable examinations (false-positives/false-negatives < 15%, fixation losses < 15%).

Patients who had a spherical equivalent lower than –6.0 diopters or eyes with an axial length greater than 26.0 mm were included in the high-myopia group. And patients who met the following inclusion criteria were enrolled consecutively in the study: (1) longer than 5-year follow-up; (2) consecutive follow-up-period RNFL photographs numbering at least five. The exclusion criteria were as follows: (1) history of intraocular surgery aside from uncomplicated cataract surgery or history of disease(s) possibly affecting the RNFL (e.g., diabetic retinopathy, ischemic optic neuropathy, retinal vein occlusion, pituitary lesions or demyelinating diseases); (2) optic disc pallor; (3) media opacity (i.e., significant cataract, asteroid hyalosis or vitreous opacity) rendering diagnostic fundus reading difficult; (4) poor-quality OCTA scan images with a signal strength less than 55; (5) pathologic myopia, as defined by a new meta-analysis classification system: 2 or higher with the presence of a plus sign (e.g., lacquer crack, myopic choroidal neovascularization, or Fuch’s spot) or posterior staphyloma^43^. In cases where both eyes were eligible for the study, one was selected randomly for inclusion.

### Definition of papillomacular bundle defect (PMBD)

**Figure 3:**
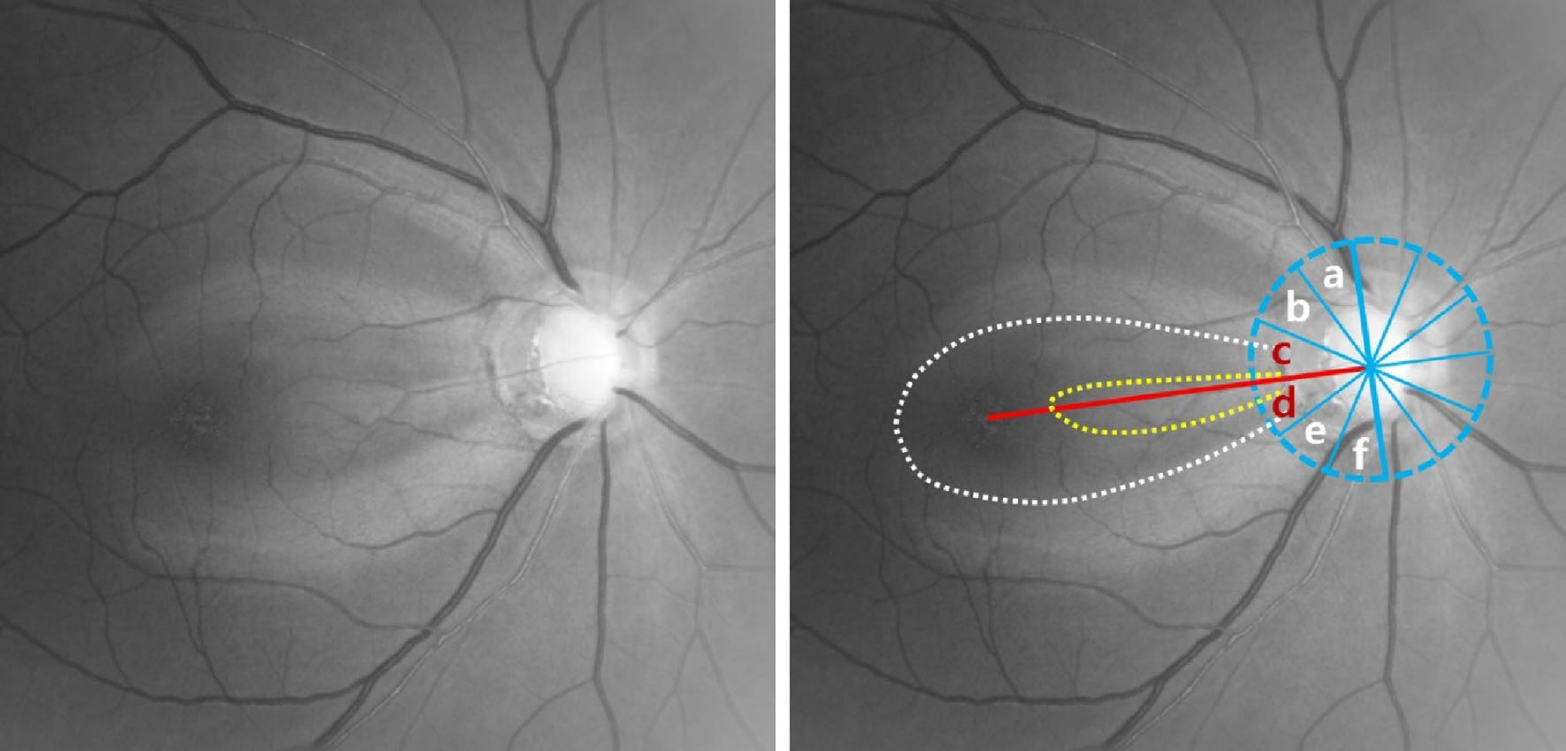
Definition of papillomacular bundle defect (PMBD). The red line is a straight line from the center of the optic disc to the foveal center and is termed the “reference line.” If a line (**a** blue solid line) is drawn running perpendicular to the reference line and passing through the center of the optic disc, and a circular line (**a** blue dotted line) is centered on the optic nerve head (ONH) to include the reference line and vertical line, the hemisphere can be divided into six equal sectors (**a**–**f** sections). Among the six sectors, the central upper and lower ones, surrounding the reference line (**c** + **d** section) from − 30 to + 30°, were defined as the papillomacular bundle (PMB) area. PMBD was declared in cases where the proximal border (yellow dotted line) of the retinal nerve fiber layer defect was located within the PMB area.

First, the definition of localized RNFL defect (RNFLD) used in this study was as follows: a well-outlined and dark wedge-shaped area within the bright striated pattern of the healthy surrounding RNFL, its tip contacting the optic disc border, on red-free RNFL photography. The PMB area was established with reference to the relevant literature^10, 14, 15^. Preparatorily, the temporal region of the optic disc was divided evenly into six sectors of 30° based on a reference line connecting the optic disc and foveal center. Then, the angular location within the − 30.0 ~  + 30.0° sectors was deemed to be the PMB area. PMBD was confirmed when the RNFLD’s proximal border was within the PMB area. The presence of PMBD and its location on RNFL photography was determined by two independent glaucoma specialists (Fig. 3). Any concomitant RNFLDs that were found outside the PMB area also were checked and analyzed.

### Disc tilt direction and parapapillary atrophy (PPA)-to-Disc-area ratio

**Figure 4:**
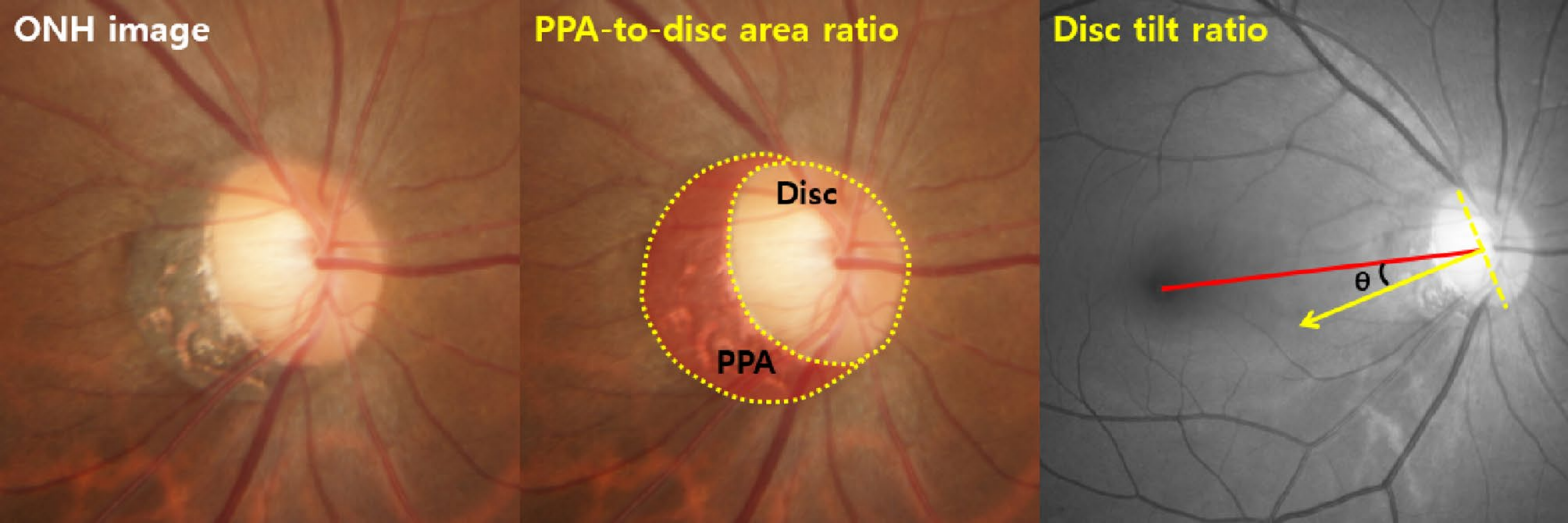
Measurement of disc parameters. The PPA and disc area (within the yellow dotted lines) were outlined manually, and the pixel area was calculated automatically using Image J software. Then the number of pixels in each area was calculated as a ratio to obtain the PPA-to-disc area ratio. The line connecting the fovea and the center of the optic disc, defined as the foveal-disc axis (red line), was used as the reference line. Disc tilt direction was defined as the deviation of the vertical axis of the disc’s longest diameter from the reference line (yellow arrow).

Color fundus photography was obtained for measurements of the optic disc and β-zone PPA. Averages of the values measured by the 2 investigators were used in the final analysis. The direction of disc tilt was defined as the deviation of the short axis of the optic disc from the reference line that connects the fovea and the center of the optic disc (Fig. 4). If the tilt direction was superior to the reference line, the angle was measured as positive, and if the direction was inferior to the reference line, the angle was measured as negative^44, 45^. The presence of β-zone PPA was defined as the region of chorioretinal atrophy with both visible sclera and choroidal vessels adjacent to the optic disc. The areas of β-zone PPA were measured using Image J (Fig. 4)^45, 46^.

### Assessment of lamina cribrosa defect (LCD)

The lamina cribrosa (LC) was imaged by SS-OCT (Topcon, Inc., Tokyo, Japan). A 3D raster scan protocol consisting of 256 × 256 A-scans was performed within 0.8 s over a 3 mm × 3 mm area that was centered on the ONH. Additionally, 12 radial-orientation raster scans (6 mm scan length, as centered on ONH) were obtained for each eye. Then, serial en face images were obtained for the 3D dataset. Both the en face images and the radial-orientation raster scan images were utilized in scrutinizing the laminar structures.

The SS-OCT image set thus obtained was independently reviewed for focal LCDs by two graders who were masked to all other information, including the presence of PMBD or its absence. The LC’s anterior surface was defined as being beneath the optic disc cup, where high reflectivity started and ended. Focal LCD was defined as any anterior laminar surface irregularity respecting the normally smooth curvilinear U- or W-shaped contour (Fig. 1C,D). To prevent false positivity, defects had to be, on en face imaging, > 100 μm in diameter and > 30 μm in depth^16^. Also, it was confirmed, by comparison of the en face imaging with the disc photography, that the candidate LCDs did not correspond to hypo-reflectivity due to vascular shadowing. If the two graders could not agree on the existence of LCD, they reviewed the evidence until reaching consensus; if consensus proved impossible, the candidate LCD was excluded from further analysis.

### Definition of central visual field defect (CVFD)

To investigate the relationship between PMBD and VF defect pattern in eyes with highly myopic glaucoma, VF defects were classified as central or non-central scotoma. We performed the 24–2 threshold testing of the Humphrey visual field in all patient and central scotoma was defined as a VF defect within the innermost 4 points, with at least one point having a P value less than 5% (Fig. 2B)^17, 18^. Non-central scotoma was defined as a VF defect in one or two hemifields outside of the innermost 4 points.

### Statistical analysis

Data herein are presented as the mean standard deviation (range) for normally distributed continuous variables and as the frequency (percentage) for categorical variables. Logistic regression models with the generalized estimating equation were used in investigating the association between central VF defect and PMBD. All of the variables for which the associations had a P value of < 0.05 in the univariate regression analysis were included in the subsequent binary multivariate regression analysis. The generalized estimating equation and the generalized linear mixed model were performed using R studio Version 1.4.1717. A P value of < 0.05 was considered statistically significant.

## Data Availability

The datasets analysed during the current study are not publicly available due to patient data privacy policy, but are available from the corresponding author (K.H.P) on reasonable request.

## Abbreviations

CVFD: Central visual field defect
DH: Disc hemorrhage
GCIPL: Ganglion cell-inner plexiform layer
IOP: Intraocular pressure
LC: Lamina cribrosa
LCD: Lamina cribrosa defect
ONH: Optic nerve head
PMBD: Papillomacular bundle defect
PPA: Parapapillary atrophy
RNFL: Retinal nerve fiber layer
SD-OCT: Spectral domain optical coherence tomography
SS-OCT: Swept-source optical coherence tomography
